# From Method-Defined Signals to Reference Measurement Procedures: Two Decades of Mass Spectrometry-Based ProGRP Quantification

**DOI:** 10.1021/acs.jproteome.6c00368

**Published:** 2026-07-02

**Authors:** Donatella Coradduzza, Emanuela Azara, Anna La Salvia, Serenella Medici, Ciriaco Carru, Troy D. Wood, Giuseppe Fanciulli

**Affiliations:** † Department of Biomedical Sciences, University of Sassari, Viale San Pietro 43/B, 07100 Sassari, Italy; ‡ Institute of Biomolecular Chemistry, National Research Council, 07100 Sassari, Italy; § National Center for Drug Research and Evaluation, National Institute of Health (ISS), 00161 Rome, Italy; ∥ Department of Chemistry and Pharmacy, University of Sassari, Via Vienna 2, 07100 Sassari, Italy; ⊥ Department of Chemistry, Natural Sciences Complex, 12292 University at Buffalo, Buffalo 14260-3000, New York, United States; # Department of Medicine, Surgery and Pharmacy, University of Sassari, Sassari 07100, Italy

**Keywords:** ProGRP, quantitative proteomics, proteoform, isotope dilution mass spectrometry, reference measurement procedure, clinical proteomics, neuroendocrine neoplasms, small cell lung cancer, metrological traceability, LC-MS/MS

## Abstract

Pro-gastrin-releasing peptide (ProGRP) is a clinically established biomarker in small cell lung cancer (SCLC) and other neuroendocrine neoplasms (NENs). Despite widespread clinical use, ProGRP measurement is challenged by low circulating concentrations and proteoform heterogeneity arising from alternative precursor processing. Current immunoassay-based methods generate operationally defined signals anchored to antibody–epitope interactions rather than chemically explicit molecular quantities, limiting result comparability across platforms. Mass spectrometry (MS) addresses these limitations through sequence-defined quantification, with the capacity to resolve sequence-distinct isoforms and, at the intact-protein level, individual proteoforms. In this Perspective, we examine two decades of MS-based ProGRP quantification as a case study for quantitative proteomics and measurement science. We trace the evolution from single-quadrupole workflows to isotope dilution strategies enabling increasingly selective and metrologically robust measurement, showing that performance gains were driven primarily by selectivity engineering and internal standardization rather than by mass analyzer advances. This trajectory progressively exposes a fundamental comparability problem: immunoassays and MS-based methods do not measure the same quantities. Resolving this discordance requires an explicit metrological framework encompassing measurand definition, traceability architecture, and reference measurement procedures (RMPs) based on protein-level isotope dilution MS. ProGRP serves as an instructive model for harmonizing quantitative protein biomarker measurements in clinical proteomics.

## Graphical abstract

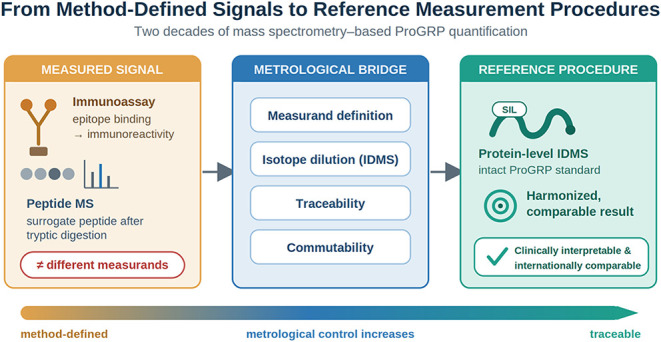

## Introduction

Pro-gastrin-releasing peptide (ProGRP) is a circulating precursor fragment of gastrin-releasing peptide (GRP) and a clinically relevant biomarker, with its most firmly established role in small cell lung cancer (SCLC), an aggressive pulmonary neuroendocrine neoplasm (NEN).^1–3^ In this setting, ProGRP has been extensively investigated for diagnostic support,^1,2,4^ disease stratification, treatment monitoring, and clinical outcome assessment.^1,2,4,5^ Beyond SCLC, increased ProGRP concentrations have also been reported in well-differentiated pulmonary NENs, and extrapulmonary NENs such as medullary thyroid carcinoma.^6,7^ As a low-abundance circulating proteoform ensemble,^8^ ProGRP represents a paradigmatic challenge for quantitative clinical proteomics: achieving the sensitivity, selectivity, and metrological rigor required for harmonized measurement across platforms and laboratories.^9–12^ Despite its widespread clinical use, the analytical measurement of ProGRP remains intrinsically challenging.^13–15^ Circulating concentrations in healthy individuals are inherently low: in a large Korean reference cohort, the upper reference limits (97.5th percentile) remained below 100 pg/mL across all age and sex strata, ranging from approximately 65 pg/mL in younger adults to 94 pg/mL in individuals aged ≥80 years, with consistently higher values in females than in males within each age group.^16,17^ These concentrations frequently approach or overlap with the lower limits of quantification of routine immunoassays, leaving a narrow and analytically demanding measurement window.^18,19^ Compounding this challenge, ProGRP does not exist as a single, chemically uniform molecular species; rather, it circulates as a heterogeneous ensemble of distinct proteoforms^20–22^ —that is, all of the different molecular forms in which the protein product of a single gene can be found, including those arising from genetic variation, alternative RNA splicing, and post-translational modifications^8,23^ —generated through alternative processing of the precursor protein.^24^

Throughout this Perspective, we use the term “proteoform” according to this accepted definition. Where bottom-up workflows distinguish sequence-distinct ProGRP variants through proteotypic peptides, we refer to isoform- or sequence-resolved measurements. The term proteoform-resolved is reserved for analytical approaches that preserve the intact molecular form since proteolytic digestion necessarily removes the combinatorial molecular information that defines a proteoform.

Together, low circulating concentrations and proteoform heterogeneity pose substantial and interrelated challenges for analytical specificity and result comparability, particularly when measurements are used to support clinical decision-making across different laboratories, platforms, and time points.^25^Currently, ProGRP is predominantly measured using immunoassay-based methods. While these assays offer high sensitivity, high throughput, and operational simplicity, their measurement principle is inherently based on antibody–epitope interactions, resulting in a signal that reflects *immunoreactivity* rather than a precisely defined molecular quantity.^26–29^ Cross-reactivity, differential epitope accessibility, and variable recognition of ProGRP proteoforms contribute to method-dependent results that are difficult to standardize.^30^ Variability in the manufacturing of critical reagents can also lead to inconsistency in the technical performance of immunoassay-based methods over time.^31,32^ As a consequence, immunoassays provide a composite and often ambiguous analytical signal, in which the exact molecular species contributing to the reported concentration remain largely undefined.^23,29^ In the context of precision medicine—where molecular specificity, traceability, and result comparability are increasingly required—this lack of clarity represents a fundamental limitation rather than a minor technical drawback.^33–36^

Mass spectrometry (MS) has emerged as a powerful alternative analytical platform capable of addressing several of these limitations.^37–40^ By targeting defined molecular species, most commonly proteotypic peptides generated after enzymatic digestion, MS-based methods offer a level of molecular specificity that is fundamentally inaccessible to immunoassays.^41,42^ Importantly, MS enables the discrimination and quantification of sequence-distinct ProGRP isoforms and, in principle, intact ProGRP proteoforms, providing access to molecular information that is masked in conventional clinical testing. From an analytical chemistry perspective, this specificity represents a decisive advantage, as it shifts the measurement from an antibody-dependent signal to a chemically defined analyte.^43,44^

However, the translation of MS-based ProGRP assays from research settings into routine clinical practice has proven to be nontrivial.^45^ Challenges related to analytical complexity, cost, and throughput have historically limited their adoption in clinical laboratories dominated by automated immunoassay platforms.^46–48^ Over the past two decades, the development of MS-based ProGRP quantification has therefore been characterized by a series of methodological and conceptual transitions aimed at overcoming these barriers. Early workflows relying on nonselective sample preparation were progressively replaced by targeted affinity-enrichment strategies, including immunocapture and molecularly imprinted polymers (MIPs), to improve sensitivity and reduce matrix effects.^49^ In parallel, advances in chromatographic separation, tandem mass spectrometry, and data acquisition strategies have significantly enhanced analytical robustness.^50^

A particularly important step in this evolution has been the introduction of stable isotope dilution approaches. The use of isotopically labeled internal standards has improved precision and repeatability and has highlighted the potential of MS to move beyond relative or method-specific quantification toward higher-order measurement principles. In this context, isotope dilution mass spectrometry (IDMS) represents more than a technical refinement; it provides a conceptual bridge between analytical chemistry and metrology, enabling explicit correction for losses, digestion efficiency, and instrumental variability.^51^

At the same time, the growing body of MS-based ProGRP studies has revealed a critical and often underappreciated issue: different analytical platforms do not necessarily measure the same quantity, even when nominally reporting “ProGRP concentration.” Immunoassays and MS-based methods are anchored to fundamentally different measurands, immunoreactivity versus defined molecular entities, and therefore generate results that may correlate but are not directly interchangeable.^52^ This discrepancy is not an analytical failure of either platform; rather, it reflects the absence of a shared metrological framework capable of linking different measurement principles to a common reference.

From a measurement-science perspective, this lack of harmonization represents a central obstacle to the clinical comparability of the ProGRP results. Without a clearly defined measurand, traceable calibration hierarchy, and commutable reference materials, numerical values obtained in different laboratories or with different platforms cannot be interpreted as equivalent, even when they are used to guide similar clinical decisions.^53^ As clinical use of ProGRP expands beyond diagnosis toward therapy monitoring and longitudinal follow-up, this limitation has become increasingly problematic.

In this perspective, we use ProGRP as a representative and instructive case study to illustrate how advances in analytical chemistry—particularly mass spectrometry—must be coupled with explicit metrological concepts to achieve true harmonization of protein biomarker measurements. Rather than providing an exhaustive methodological review, we focus on the conceptual evolution of MS-based ProGRP quantification, highlighting key transitions from peptide-centric workflows to isotope dilution strategies. We argue that the development of reference measurement procedures (RMPs), potentially based on intact-protein standards and clearly defined measurands, represents the logical next step toward internationally comparable and clinically interpretable ProGRP measurements.^54^ More broadly, the challenges and solutions discussed here are not unique to ProGRP but exemplify a general problem in protein biomarker quantification at the interface between analytical chemistry and clinical practice.^55^

## Scope and Perspective

In this Perspective, we critically examine the conceptual evolution of mass spectrometry-based approaches for ProGRP measurement, not as a chronological method-by-method review, but as a case study in analytical design and metrological decision-making. We analyze how successive innovations in sample preparation, affinity enrichment, and isotope dilution strategies have progressively improved analytical performance, while at the same time exposing fundamental limitations related to measurand definition, traceability, and result comparability. Rather than identifying a single “best” method, we focus on clarifying why different analytical pathways yield noninterchangeable results and on outlining the key metrological requirements—particularly the development of reference measurement procedures—that must be addressed for MS-based ProGRP quantification to support harmonized clinical interpretation.

## Evidence Base and Selection Rationale

A structured literature search was performed through focused examination of the literature on mass spectrometry-based quantification of pro-gastrin-releasing peptide (ProGRP). Relevant studies were identified through searches of PubMed, Scopus, and Web of Science using combinations of keywords covering ProGRP, LC–MS, quantitative mass spectrometry, isotope dilution, and biomarker measurement. The search was last updated in March 2026 and was intended to capture representative, method-defining studies. Rather than aiming for exhaustive coverage, the intent was to identify methodologically representative and conceptually influential contributions that illustrate how MS-based ProGRP measurement has evolved over time and how different analytical choices have shaped the reported results.

The selected studies share a common focus on quantitative determination of ProGRP in clinically relevant matrices, predominantly human serum or plasma, and collectively span the major analytical strategies that have emerged over the past two decades. These include differences in sample preparation philosophy, affinity-based enrichment (both antibody-dependent and antibody-free), digestion workflows, internal standardization approaches, and mass-spectrometric detection modes. Studies lacking a quantitative component, relying on nonhuman or artificial matrices, or addressing ProGRP only marginally were considered informative for background but were not central to the analytical framework developed here.

We treat the selected papers as case studies in analytical and metrological decision-making rather than as entries in a performance ranking. For each work, attention was given not only to reported figures of merit–such as sensitivity, precision, and accuracy–but also to the implicit definition of the measurand, the level at which isotope dilution was applied, and the extent to which preanalytical and procedural losses were controlled or acknowledged. This approach allows methodological similarities and differences to be interpreted in terms of their impact on result comparability, rather than solely on analytical performance. To support this discussion, key features of the selected workflows are summarized in comparative tables. Table 1 highlights the principal methodological pathways, focusing on sample preparation, enrichment strategy, and MS detection. Table 2 brings together reported analytical performance characteristics, facilitating a qualitative comparison across approaches while emphasizing their underlying assumptions. Detailed instrumental conditions are provided in Table S1 (Supporting Information) for completeness, but the central emphasis of this Perspective lies in the conceptual implications of these methods rather than in exhaustive technical parameter listings.

**Table 1:** Analytical Workflow, Implied Measurand, and Metrological Gap^a^

study	sample prep/enrichment	digestion	internal std.	implied measurand	MS platform	metrological gap
Winther et al.	Protein precipitation/No enrichment	Trypsin, offline	None	Total ProGRP (single surrogate peptide)	Single-quadrupole LC-MS (SIM)	No SIL, undefined proteoform contribution, no traceability
Winther et al.	Immunocapture 96-well + SPE	Trypsin, offline	Structural analog (unlabeled)	Total ProGRP via common peptide, immunocapture-defined	Triple quadrupole LC-MS/MS (SRM)	Partial correction for losses; antibody-dependent selectivity; no protein-level IDMS
Winther et al.	Immunocapture + comprehensive ISTD	Trypsin, offline	SIL peptide (added postdigest)	Total ProGRP, immunocapture-framed, improved precision	Triple quadrupole LC-MS/MS (SRM)	No correction for predigest losses; peptide-level traceability only
Torsetnes et al.	Immunocapture(isoform-resolved)	Trypsin, offline	SIL peptides(per isoform)	Individual ProGRP isoforms (1, 2, 3) chemically resolved	Triple quadrupole LC-MS/MS (SRM)	Isoform-specific; no harmonized clinical reference; requires clinically validated cut-offs per isoform
Torsetnes et al.	Multiplexed immunocapture (ProGRP + NSE)	Trypsin, offline	SIL peptides (multiplex)	Simultaneous isoform-resolved ProGRP + NSE molecular panel	Triple quadrupole LC-MS/MS (MRM)	Measurand multiplicity; different clinical decision limits required
Rossetti et al.	MIP postdigest enrichment (antibody-free)	Trypsin, offline	SIL peptide	ProGRP-derived peptide, MIP-captured (synthetic affinity)	Triple quadrupole LC-MS/MS (SRM)	Peptide-level capture; matrix-dependent recovery not fully controlled
Levernæs et al.	Online nanoLC immunocapture, integrated digestion	Immobilized trypsin, online	SIL peptide	Total ProGRP (operationally defined by online immunocapture)	nanoLC-MS/MS (SRM)	Automation reduces variability; peptide-level traceability; no intact-protein standard
Li et al.	Immunoaffinity vs antibody-free IDMS (parallel comparison)	Trypsin, two configurations	SIL peptides; IDMS-oriented approach	Defined ProGRP peptide(s); explicit comparison of two measurand anchors	High-resolution MS (Orbitrap, HR-PRM)	Best current metrological control; still peptide-based; intact-protein IDMS not yet implemented

a The “Implied measurand” column reflects the quantity operationally defined by each workflow rather than an explicitly stated target in the original publication. “Metrological gap” summarizes the main limitations preventing direct qualification as a reference measurement procedure.

**Table 2:** Reported Analytical Performance and Comparability Context^a^ ^,^ ^b^

study	matrix	LOD	LOQ	dynamic range	CV (%) intraday	CV (%) interday	bias vs Immunoassay
Winther et al.	Human serum	13.9 pg on-column	NR	NR	∼10–15	NR	Not assessed
Winther et al.	Human serum	∼0.3 ng/mL	∼1 ng/mL	1–200 ng/mL	<10	<15	Systematic bias, direction NR
Winther et al.	Human serum	NR	∼0.5 ng/mL	0.5–100 ng/mL	<8	<12	Not assessed directly
Torsetnes et al.	Human serum	∼0.1 ng/mL (per isoform)	∼0.3 ng/mL	0.3–100 ng/mL	<8	<12	Compared with ELISA; significant systematic difference
Torsetnes et al.	Human serum	∼0.1 ng/mL	∼0.3 ng/mL	0.3–100 ng/mL	<7	<10	Bias present; attributed to different measurands
Rossetti et al.	Human serum	NR	∼1 ng/mL	1–100 ng/mL	<12	NR	Not formally assessed
Levernæs et al.	Human serum	∼0.2 ng/mL	∼0.5 ng/mL	0.5–50 ng/mL	<7	<10	Not formally assessed
Li et al.	Human plasma	∼0.1–0.2 ng/mL	∼0.3 ng/mL	0.3–100 ng/mL	4–6	5–8	Systematic bias ∼30–50% vs immunoassay; interpreted as measurand difference

a Reported LOD and LOQ values are not directly comparable across studies due to differences in units, injected volume, digestion yield, recovery, and calibration strategy. Values are presented as originally reported to preserve methodological context. NR = not reported.

b LOD/LOQ definitions: LOD is the lowest analyte amount or concentration producing a signal reliably distinguishable from background (commonly S/N ≈ 3). LOQ is the lowest concentration measurable with acceptable precision and trueness (commonly S/N ≈ 10 or CV ≤ 20%). The exact criterion differs among studies, and values are reported as originally published; therefore, direct comparison across studies should be interpreted cautiously.

## Conceptual Evolution of MS-Based ProGRP Quantification

The ProGRP literature offers an instructive case study of protein quantification by mass spectrometry, demonstrating that analytical performance is rarely governed by the mass analyzer alone.^15,56^ Across two decades of method development—spanning single-quadrupole and triple-quadrupole LC-MS platforms, and more recently high-resolution accurate-mass (HRAM) platforms (e.g., Orbitrap-based systems)—the most consequential improvements have instead originated upstream, at the level of analytical design.^57–59^ Specifically, gains in performance have been driven by changes in selectivity architecture (from nonselective protein precipitation to antibody-based and synthetic affinity enrichment), by refinement of the analytical target (from surrogate peptides intended to represent “total ProGRP” to isoform-resolved and sequence-resolved peptide readouts), and by evolution of internal standardization strategies (from no internal standard, to structural analogs, and, ultimately, to isotope dilution with explicit correction of process losses).^60,61^

When interpreted as a measurement-science trajectory rather than as a chronological sequence of instruments, MS-based ProGRP assays reveal a consistent pattern: improvements in sensitivity and precision—achieved across diverse platforms and workflows—have coincided with an increasing recognition that different analytical configurations operationally quantify different measurands.^62,63^ As a consequence, numerical results obtained on different MS platforms (e.g., immunocapture-LC-MS/MS on triple quadrupoles versus antibody-free IDMS workflows on high-resolution systems) may correlate yet remain noninterchangeable without a shared measurand and traceability framework.^64,65^ In this sense, the field has progressed from addressing a feasibility question (“Can ProGRP be detected and quantified by MS in clinical matrices?”) to confronting a comparability problem (“What quantity is being measured, and under what conditions can that quantity be transferred across platforms, laboratories, and clinical contexts?”).^66^

Table 1 summarizes selected milestone MS-based ProGRP workflows, emphasizing how analytical design choices implicitly define the measurand and determine the degree of metrological control that is achieved.

### Sensitivity is Mainly Driven by Selectivity Engineering

Early MS approaches established feasibility but also demonstrated that the limiting factor for clinically relevant ProGRP measurement was not MS sensitivity *per se*.^67,68^ Instead, the dominant bottlenecks were matrix complexity, low analyte abundance, and poorly controlled recovery across multistep workflows.^69^ The initial single-quadrupole strategy reported by Winther et al. reached an LOD of 13.9 pg on-column, a level compatible primarily with markedly elevated concentrations and therefore insufficient for broad clinical use, where decision-making often occurs at lower concentration ranges.^44^ Conceptually, these early studies clarified a point that remains valid today: for trace-level protein biomarkers, effective analytical sensitivity is often governed upstream of the detector, i.e., by the ability to isolate the relevant molecular signal from the biological matrix.^70^

A decisive turning point was the adoption of affinity-based enrichment, which increased effective selectivity and reduced ion suppression. Immunocapture in a 96-well format coupled with solid-phase extraction^71,72^ moved MS-based readouts into a clinically relevant range (LOQ on the order of subng/mL), representing a practical breakthrough—a step change in achievable sensitivity in clinical matrices that could not be attained through chromatographic or instrumental optimization alone.^72^ In the ProGRP case, affinity enrichment functioned less as a “convenience step” and more as a measurement-enabling transformation in selectivity architecture that materially increased effective sensitivity, converting an analytically underpowered workflow into one that could realistically interface with clinical concentration ranges.^73^

At the same time, the exploration of synthetic affinity materials introduced a conceptually important alternative: selectivity engineered through chemistry, rather than immunorecognition. Molecularly imprinted polymers (MIPs) for postdigest enrichment illustrate how peptide-level capture can offer operational robustness and potentially scalable manufacturing.^49^ Yet, these antibody-free approaches also highlight an important nuance for ProGRP: enrichment at the peptide level can simplify workflows but does not automatically meet the sensitivity demands imposed by low endogenous concentrations and sample losses in real matrices.^74^ Consequently, the apparent sensitivity trajectory should be interpreted as qualitative evidence of methodological progress rather than as a strict quantitative ranking across heterogeneous reporting frameworks.^75^ These values cannot be compared across publications without harmonizing key assumptions such as injected volume,^76^ extraction and enrichment recovery, digestion yield, matrix-dependent ion suppression, and calibration strategy.^77,78^

### Isoform-Resolved and Sequence-Defined Measurements Change What “ProGRP” Means Analytically

A more profound shift has been the transition from workflows reporting “total ProGRP,” often through a single common proteotypic peptide, to isoform-resolved measurement strategies that distinguish sequence-defined ProGRP variants.^79,80^ Isoform-resolved workflows^81^ demonstrated that ProGRP circulates as a heterogeneous ensemble and that the relative abundance of isoforms varies across patients.^43^ This observation is not merely a biological detail; it directly impacts measurement comparability because “total ProGRP” values become dependent on (i) which molecular species contribute to the assay signal, and (ii) how the method operationally defines and captures those species.^82^

Isoform specificity is analytically powerful because it replaces a method-defined target (“ProGRP”) with sequence-defined peptide readouts. From the perspective of clinical implementation, however, proteoform specificity introduces a crucial question: Which measurand is clinically intended (total immunoreactivity, a defined peptide surrogate, or intact proteoform-resolved ProGRP)? A single surrogate peptide intended to represent “total ProGRP” may not behave as a stable proxy if proteoform composition varies markedly across individuals or disease states. In other words, improved molecular resolution makes explicit that what was previously treated as a single analyte may be an analyte family.

The subsequent multiplexed format integrating ProGRP isoforms with NSE quantification illustrates a second advantage of MS: the capacity to expand from single-analyte quantification to molecular panels within one analytical framework.^43,83^ Yet multiplexing also makes the metrological issue more visible: as analytical readouts become richer and more molecularly defined, differences between MS and immunoassay results are less likely to be “fixed” by incremental optimization and more likely to reflect a fundamental difference in what is being measured.^84^

### Robustness Improved with Isotope Dilution, but Comparability Remains a Metrological Issue

Across the timeline, reported reproducibility improves substantially, consistent with progressive adoption of internal standardization and better-controlled sample preparation. Early procedures commonly reported RSD values on the order of ∼10% or higher, reflecting cumulative variability contributed by sample handling, digestion efficiency, and matrix effects. Later assays using stable isotope-labeled peptides often reach within-laboratory precision compatible with clinical implementation, and the most recent work achieves RSD values in the ∼4–6% range while explicitly comparing immunoaffinity and antibody-free IDMS-oriented workflows.^85^

Here, it is critical to separate two notions that are often conflated: analytical robustness and intermethod comparability. Isotope dilution can greatly improve robustness within a given workflow by normalizing instrumental response and compensating for losses at specific points in the process.^86^ Nevertheless, comparative studies consistently show systematic differences between MS and immunoassays. Observed systematic differences are consistent with different measurands (epitope-defined immunoreactivity versus sequence-defined MS targets) rather than with random analytical error.^31,87^ This distinction is essential for a metrology-forward interpretation: a bias does not necessarily indicate that one method is “wrong” but may indicate that the methods are anchored to different quantities. From a measurement-science standpoint, such differences are expected in the absence of (i) an agreed measurand definition, (ii) a traceability chain linked to commutable materials, and (iii) reference measurement procedures capable of harmonizing calibration across platforms.^88,89^

### Sample Volume and Throughput: from Research-Only to Clinical Feasibility

Practical constraints—sample volume and throughput—also evolved meaningfully. Early methods commonly required large volumes (up to ∼1 mL) and long end-to-end cycle times when digestion and offline steps were included. More recent strategies reduced sample volume (e.g., ∼0.2 mL) and introduced automation through online enrichment and integrated digestion, reducing hands-on time and improving throughput.^90^ These developments are not merely operational conveniences: reducing sample volume can expand applicability to serial monitoring and resource-limited clinical scenarios, while automation reduces operator-dependent variability—an underappreciated contributor to interlaboratory differences.

At the same time, ProGRP remains a revealing case study of the persistent tension between analytical ambition and clinical practicality. Even when workflows become operationally efficient, their results remain method-defined, unless embedded within a broader metrological framework that addresses measurand definition, traceability, and commutability. Thus, operational feasibility is necessary for adoption but insufficient for harmonization.

The methodological trajectories summarized above are supported by the comparative tables. Table 1 captures the major workflow architectures (sample preparation, enrichment, digestion, internal standardization, and platform), while Table 2 consolidates the key reported performance parameters (LOD/LOQ, dynamic range, precision, and bias where available). Supporting Table S1 provides LC-MS configuration details for completeness and reproducibility. Reported cycle times are not standardized across publications and may or may not include digestion/incubation steps, and should therefore be interpreted as indicative rather than strictly comparable metrics. In this Perspective, the tables are intended to anchor the conceptual discussion and to make explicit how specific workflow decisions translate into different analytical targets and different degrees of metrological control, rather than to serve as a ranking of methods. Because measurands and workflow architectures differ, reported performance metrics should be interpreted as context-dependent rather than directly comparable across studies.

## Discussion

### Measurand and Traceability Framework

A central premise of this Perspective is that disagreement among ProGRP results cannot be resolved without first defining the measurable. In current practice, immunoassays and mass spectrometry methods generate analytically valid results that are nonetheless anchored to different quantities: epitope-dependent immunoreactivity on the one hand and sequence-defined molecular entities on the other. In MS-based workflows, the operational measurand is most often the amount-of-substance concentration of a specific ProGRP-derived peptide released by enzymatic digestion under defined conditions rather than the intact circulating protein itself. This distinction is metrological, not semantic, because traceability, commutability, and result transferability can be established only once the measurand is explicitly defined. Framed in this way, MS should be viewed not simply as an alternative detection technology but as an enabling platform for constructing reference measurement procedures based on isotope dilution principles, provided that calibration hierarchies, preanalytical control, and commutable reference materials are addressed in a coherent measurement system.

### Measurand: from Operational Signals to Chemically Defined Quantities

The evolution of MS-based ProGRP assays has progressively revealed that analytical performance alone cannot resolve the long-standing discordance between measurement platforms unless the measurand itself is explicitly defined. Early MS methods implicitly adopted the same conceptual target as immunoassays—“total ProGRP”—while in practice, quantifying a single proteotypic peptide generated under specific digestion conditions. As analytical selectivity increased, particularly with the advent of affinity enrichment and isoform-resolved workflows, it became evident that ProGRP is not a single molecular entity but a heterogeneous ensemble of circulating proteoforms.

Isoform-resolved quantification, first demonstrated by Torsetnes et al., represents a conceptual shift: MS can report chemically defined targets rather than a composite immunoreactivity signal. From a measurement-science perspective, this is a strength, as it replaces an ill-defined biomarker with a sequence-resolved target. At the same time, it exposes a critical ambiguity: “total ProGRP” values are method-dependent constructs whose numerical meaning varies with proteoform composition, antibody specificity, and digestion strategy. Consequently, numerical disagreement between MS and immunoassays should be interpreted as evidence of different measurable quantities, not analytical failure. This realization has direct implications for the clinical translation. If MS-based assays continue to report peptide-derived surrogates, then the measurand must be declared as such (e.g., amount-of-substance concentration of a defined ProGRP-derived peptide under specified digestion conditions). Alternatively, if intact ProGRP proteoforms are to be the clinically relevant targets, analytical strategies must evolve accordingly. In either case, measurand definition is not an academic exercise but the foundation upon which traceability, comparability, and clinical interpretation depend. As reviewed for top-down proteomics more broadly, intact-protein characterization can resolve and quantify biomarker proteoforms directly, rather than through surrogate peptides.^91^ This has been demonstrated for cardiac troponin I, whose disease-associated phosphorylation proteoforms were resolved by top-down MS, revealing clinically relevant molecular heterogeneity inaccessible to peptide-based assays;^92^ the same proteoform-resolved paradigm is directly transferable to ProGRP, reinforcing the case for protein-level reference measurement procedures.^91,92^

### Traceability and IDMS: Moving from Precision to Reference Measurement

Improvements in reproducibility across the ProGRP literature closely follow the adoption of increasingly rigorous internal standardization strategies. Early workflows lacking internal standards exhibited substantial variability, while the introduction of structural analogs partially corrected for sample handling losses. The widespread adoption of stable isotope-labeled (SIL) peptides marked a major step toward improved control of key sources of variability, enabling compensation for digestion efficiency, ion suppression, and instrumental variability. However, while SIL peptides substantially improve within-method precision, they do not, by themselves, establish traceability. Peptide-based isotope dilution anchors quantification to an operational surrogate rather than to the intact biomarker.

This distinction becomes critical when considering interplatform comparability and the development of reference measurement procedures (RMPs). Recent work explicitly comparing immunoaffinity and antibody-free IDMS-oriented workflows highlights both the promise and the limitation of current approaches: precision is excellent, yet systematic bias relative to immunoassays persists. From a metrological standpoint, this bias should not be framed as greater or lesser “accuracy.” Instead, it reflects fundamentally different measurement principles—epitope-dependent immunoreactivity versus sequence-defined molecular quantification. Accuracy can only be asserted relative to an agreed reference system; in the absence of higher-order reference materials and an agreed measurand, such accuracy claims remain provisional. Progressing from peptide-based IDMS to protein-level IDMS, supported by intact isotopically labeled ProGRP standards, represents a logical next step toward establishing traceable RMPs capable of harmonizing results across MS platforms and, ultimately, across technologies.

### Commutability and Implementation: Where Analytical Excellence Meets Reality

Commutability is the property of a reference material, whereby the relationship between results obtained by different measurement procedures for that material is equivalent to the relationship observed for authentic patient samples. Lack of commutability may introduce procedure-dependent bias and compromise calibration transfer, even when individual measurement procedures exhibit an acceptable analytical performance.

Even analytically and metrologically sound methods fail to achieve clinical impact if they cannot be implemented in a commutable and operationally realistic manner. The ProGRP case exemplifies this tension. Despite impressive gains in sensitivity through affinity enrichment—whether antibody-based or synthetic—translation into routine clinical laboratories remains limited. Factors such as material reproducibility, cost, availability of standardized kits, and compatibility with high-throughput workflows continue to constrain adoption. Synthetic receptors, including molecularly imprinted polymers (MIPs), offer a promising route toward improved robustness and scalability. Their chemical stability, resistance to degradation, and potential for highly reproducible manufacturing may mitigate several limitations associated with biological antibodies, including lot-to-lot variability and long-term reagent instability. These characteristics make synthetic affinity reagents attractive candidates for future metrologically controlled MS workflows.

Parallel advances in automation, online enrichment, and miniaturized systems further reduce the hands-on time and operator-dependent variability. Nevertheless, throughput remains constrained by enzymatic digestion and incubation steps, positioning MS more realistically as a confirmatory or specialized diagnostic tool rather than a universal screening replacement for immunoassays in the near-term.

Crucially, commutability is not achieved through automation alone. Preanalytical variables—sample handling time, temperature, freeze–thaw cycles, adsorption to surfaces, and proteoform stability—exert a decisive influence on MS-based readouts. Without harmonized preanalytical SOPs and shared stability data, even highly controlled analytical workflows remain method-defined. Thus, commutability must be addressed as an integrated property of the entire measurement chain, from sample collection to result reporting.

Finally, clinical implementation requires an alignment between analytically defined quantities and clinical decision-making. MS-based assays often report lower ProGRP values than immunoassays, consistent with differences in the measurand definition and analytical specificity. This necessitates the development of MS-defined clinical decision limits, derived from outcome-based studies rather than transplanted from the immunoassay literature. Without such recalibration, the analytical advantages of MS cannot be fully translated into clinical value (Figure 1).

**Figure 1:**
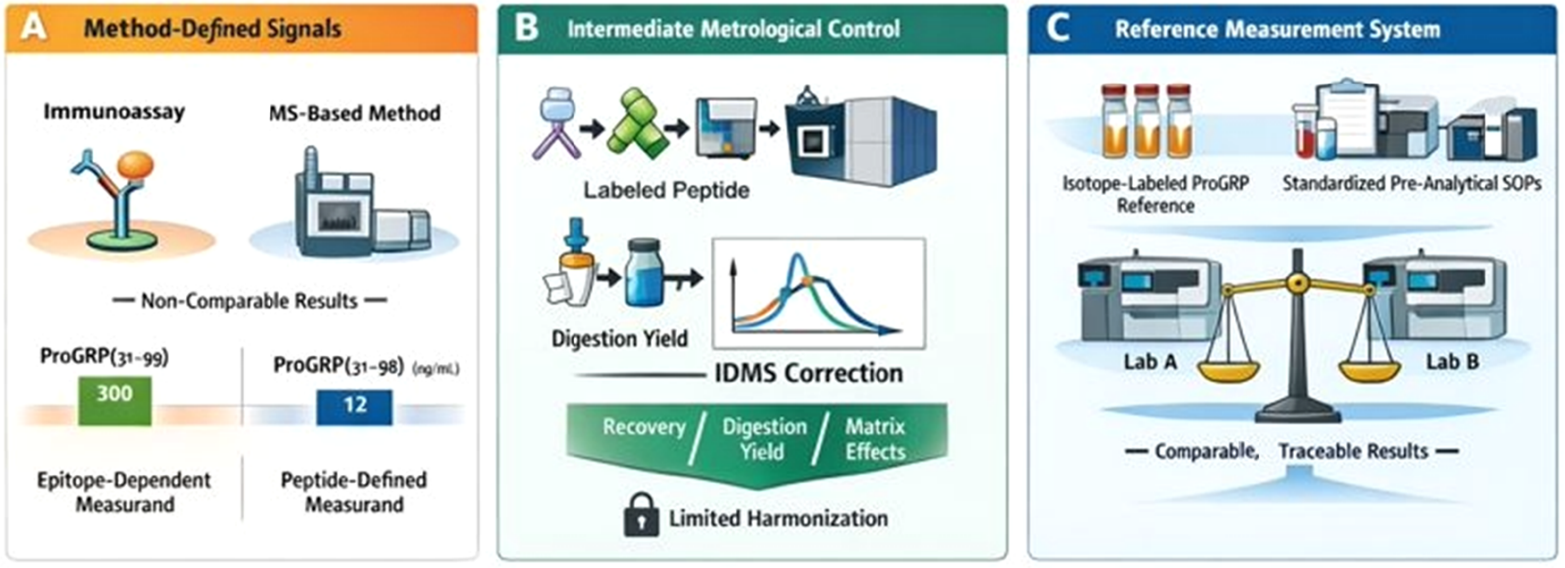
From method-defined ProGRP signals to reference measurement procedures. Panel (A): current landscape showing immunoassays and MS-based methods anchored to different measurands. Panel (B): role of explicit measurand definition and IDMS. Panel (C): target reference measurement system with protein-level IDMS, commutable reference materials, and harmonized preanalytical procedures.

### Perspective Takeaway

Viewed through the lens of measurement science, ProGRP is not simply a challenging biomarker but a revealing model system. It demonstrates that sensitivity, specificity, and precision—while necessary—are insufficient to achieve harmonized clinical measurement. The remaining barriers are metrological: explicit measurand definition, traceability to higher-order references, and commutability across platforms. Addressing these dimensions transforms MS from an alternative analytical technique into a practical platform for building reference measurement procedures and traceability chains, with implications extending well beyond ProGRP itself.

## Conclusions

The analytical evolution of pro-gastrin-releasing peptide (ProGRP) quantification offers a clear illustration of how progress in mass spectrometry (MS) progressively shifts the central challenge from analytical performance to measurement science. Over the past two decades, MS-based approaches have advanced from proof-of-concept studies to highly selective, sequence-defined, and isoform-resolved assays supported by isotope dilution strategies. These developments unequivocally demonstrate that MS can meet—and in several respects surpass—the technical requirements for sensitivity, specificity, and precision demanded by clinical applications such as small cell lung cancer (SCLC).

At the same time, the evidence synthesized in this Perspective makes it clear that analytical capability alone is insufficient to achieve harmonized clinical measurement. The persistent discrepancies observed between MS-based methods and immunoassays should not be interpreted as analytical inadequacy but rather as the consequence of fundamentally different measurands and the lack of traceability chains linking routine measurements to higher-order references. In this context, MS should not be regarded merely as an alternative analytical technology but as an enabling platform for the construction of reference measurement procedures (RMPs) consistent with internationally recognized metrological principles.

In the context of the International Federation of Clinical Chemistry and Laboratory Medicine (IFCC) and the Joint Committee for Traceability in Laboratory Medicine (JCTLM) standardization principles, ProGRP currently lacks the essential components required for full standardization. A formally defined measurand, commutable reference materials, and validated RMPs capable of supporting traceability across platforms are still missing. Peptide-based isotope dilution MS represents a crucial intermediate step in this trajectory, providing robust internal correction and improved within-method precision. Nevertheless, such approaches remain operational by nature and cannot, on their own, establish traceability to an intact biomarker. Progression toward protein-level IDMS, supported by isotopically labeled intact ProGRP proteoforms, therefore, represents a promising future direction for the development of higher-order reference systems suitable for international recognition.

Equally critical is the recognition that commutability cannot be treated as a secondary analytical consideration. Preanalytical variables—including sample handling, temperature, freeze–thaw cycles, adsorption effects, and proteoform stability—exert a decisive influence on MS-based readouts. Without harmonized preanalytical standard operating procedures and shared stability data, even rigorously validated analytical workflows cannot ensure result transferability across laboratories, undermining the very foundations of IFCC-endorsed reference systems.

Taken together, the body of evidence supports a coherent metrological roadmap in which explicit measurand definition, strategic selection of IDMS architecture, development of higher-order reference materials, standardization of preanalytical conditions, and multilaboratory validation converge toward the establishment of robust RMPs. Within such a framework, alignment with clinical interpretation naturally follows, enabling the derivation of MS-defined decision limits that can be traced to reference systems rather than inherited from legacy immunoassays.

In this light, MS-based ProGRP quantification should not be viewed as a disruptive replacement of immunoassays but as the metrological backbone upon which harmonized calibration, intermethod alignment, and clinically interpretable results can be built. While ProGRP serves as the motivating case study, the conceptual framework outlined here is broadly applicable to many low-abundance protein biomarkers currently affected by intermethod discordance. More broadly, it illustrates how analytical chemistry, when integrated with laboratory medicine and metrology, can enable the transition from method-defined signals to internationally traceable clinical measurements.

## Supplementary Material



## Data Availability

No new experimental data were generated for this study. All data discussed are derived from previously published works cited in the reference list.

## Notes

Detailed LC–MS/MS instrumental configurations for all MS-based ProGRP quantification methods discussed in this Perspective (Table S1) (PDF)
