# Supplementary material for: From Method-Defined Signals to Reference Measurement Procedures: Two Decades of Mass Spectrometry-Based ProGRP Quantification
Source: J Proteome Res. 2026 Jul 2;25(8):3806–16. doi: 10.1021/acs.jproteome.6c00368 (PMC13459549; doi:10.1021/acs.jproteome.6c00368)
Supplement: Supplementary file 1 [file pr6c00368_si_001.pdf]

Supporting Information

From Method-Defined Signals to Reference Measurement Procedures: Two Decades of Mass Spectrometry-Based ProGRP Quantification

Donatella Coradduzza[*,1] Emanuela Azara[2], Anna La Salvia[3], Serenella Medici[4], Ciriaco Carru[1], Troy D. Wood[5], Giuseppe Fanciulli[6]

1 Department of Biomedical Sciences, University of Sassari, Viale San Pietro 43/B, 07100, Sassari, Italy.
2 Institute of Biomolecular Chemistry, National Research Council, 07100, Sassari, Italy.
3 National Center for Drug Research and Evaluation, National Institute of Health (ISS), 00161, Rome, Italy.
4 Department of Chemistry and Pharmacy, University of Sassari, Via Vienna 2, 07100 Sassari, Italy.
5 Department of Chemistry, Natural Sciences Complex, University at Buffalo, Buffalo, 14260-3000, New York, USA.
6 Department of Medicine, Surgery and Pharmacy, University of Sassari, Sassari, 07100, Italy.

***Corresponding Author:** Donatella Coradduzza. Email: dcoradduzza@uniss.it

Table of Contents

Table S1. Detailed LC–MS/MS instrumental configurations S-2

Table S1. Detailed LC–MS/MS instrumental configurations for MS-based ProGRP quantification methods discussed in this Perspective. NR = not reported; SIM = selected ion monitoring; SRM = selected reaction monitoring; MRM = multiple reaction monitoring; HR-PRM = high-resolution parallel reaction monitoring; IMER = immobilized enzyme reactor; IA = immunoaffinity; IDMS = isotope dilution mass spectrometry; RAM = restricted access material.

Table S1 (Supporting Information). Detailed LC–MS Instrumental Configurations

Study	LC Column	Mobile Phase	Gradient / Flow	MS Mode	Monitored Transitions	Cycle Time / Notes
Winther et al., 2007	C18 RP (2.1×50 mm, 3 µm)	A: 0.1% FA in H_2O; B: 0.1% FA in ACN	5–95% B, 10 min; 200 µL/min	SIM	Protonated precursor $[M+H]^+$	~30 min total; offline digestion ~12 h
Winther et al., 2009a	C18 RP (2.1×100 mm, 3 µm)	A: 0.1% FA in H_2O; B: 0.1% FA in ACN	5–60% B, 15 min; 200 µL/min	SRM (triple quad)	2–3 transitions/peptide	~45 min; immunocapture 96-well, overnight digestion
Torsetnes et al., 2013	C18 RP (2.1×50 mm, 1.7 µm)	A: 0.1% FA; B: ACN/FA	5–45% B, 8 min; 400 µL/min	SRM (triple quad)	2–4 transitions/isoform	~60 min total; isoform-resolved immunocapture
Torsetnes et al., 2014	C18 RP (2.1×50 mm, 1.7 µm)	A: 0.1% FA; B: ACN/FA	Multiplexed; 400 µL/min	MRM (triple quad)	ProGRP + NSE multiplex panel	~90 min incl. multiplexed immunocapture; simultaneous ProGRP + NSE
Rossetti et al., 2014	C18 RP nano (75 µm×150 mm)	A: 0.1% FA; B: ACN	5–50% B, 40 min; 300 nL/min	SRM (triple quad)	3 transitions; MIP-captured peptide	~120 min; MIP batch SPE, offline digestion
Levernæs et al., 2018	Monolithic nanoLC (immobilized Ab)	A: 0.1% FA; B: ACN	Online enrichment + gradient; 300 nL/min	SRM (triple quad)	2–3 transitions; online integrated digestion	~45 min total run; 0.2 mL sample; online IMER
Li et al., 2025	C18 RP (2.1×100 mm, 1.8 µm)	A: 0.1% FA; B: ACN/FA	5–60% B, 12 min; 300 µL/min	HR-PRM (Orbitrap)	High-res transitions; parallel IA and IDMS workflows	~60–90 min; comparative dual-workflow design

Abbreviations: 2D-LC, two-dimensional liquid chromatography; HRAM, high-resolution accurate mass; HR-PRM, high-resolution parallel reaction monitoring; IDMS, isotope dilution mass spectrometry; LC, liquid chromatography; LOD, limit of detection; LOQ, limit of quantification; MIP, molecularly imprinted polymer; NEN, neuroendocrine neoplasm; NSE, neuron-specific enolase; RAM, restricted access material; SCLC, small cell lung cancer; SIL, stable isotope-labeled; SIM, selected ion monitoring; SPE, solid-phase extraction; SRM, selected reaction monitoring.

Note: Cycle times may include or exclude offline digestion/incubation steps depending on the original publication; they should be interpreted as indicative rather than directly comparable metrics.
